# Morpho-Physiological, Biochemical, and Genetic Responses to Salinity in *Medicago truncatula*

**DOI:** 10.3390/plants10040808

**Published:** 2021-04-20

**Authors:** Sabrine Hdira, Loua Haddoudi, Mohsen Hanana, Irene Romero, Asma Mahjoub, Hatem Ben Jouira, Ndiko Ludidi, Maria Teresa Sanchez-Ballesta, Chedly Abdelly, Mounawer Badri

**Affiliations:** 1 Laboratory of Extremophile Plants, Centre of Biotechnology of Borj Cedria, B.P. 901, Hammam-Lif 2050, Tunisia; hdirasabrina@gmail.com (S.H.); loua.haddoudi@outlook.fr (L.H.); punto80@yahoo.com (M.H.); Asma_inrat@yahoo.fr (A.M.); hatembj@gmail.com (H.B.J.); abdelly.chedly@gmail.com (C.A.); 2 Faculty of Mathematical, Physical and Natural Sciences of Tunis, Campus Universitaire El-Manar, University of Tunis El Manar, El Manar 2092, Tunisia; 3 Laboratory of Biotechnology and Postharvest Quality Institute of Food Science, Technology and Nutrition (ICTAN-CSIC), Jose Antonio Novais, 10, 28040 Madrid, Spain; irene.romero@ictan.csic.es (I.R.); mballesta@ictan.csic.es (M.T.S.-B.); 4 Plant Biotechnology Research Group, Department of Biotechnology, University of the Western Cape, Robert Sobukwe Road, Bellville 7530, South Africa; nludidi@uwc.ac.za; 5 DSI-NRF Centre of Excellence in Food Security, University of the Western Cape, Robert Sobukwe Road, Bellville 7530, South Africa

**Keywords:** *Medicago truncatula*, morpho-physiological traits, biochemical parameters, AP2/ERF family, salt stress

## Abstract

We used an integrated morpho-physiological, biochemical, and genetic approach to investigate the salt responses of four lines (TN1.11, TN6.18, JA17, and A10) of *Medicago truncatula*. Results showed that TN1.11 exhibited a high tolerance to salinity, compared with the other lines, recording a salinity induced an increase in soluble sugars and soluble proteins, a slight decrease in malondialdehyde (MDA) accumulation, and less reduction in plant biomass. TN6.18 was the most susceptible to salinity as it showed less plant weight, had elevated levels of MDA, and lower levels of soluble sugars and soluble proteins under salt stress. As transcription factors of the APETALA2/ethylene responsive factor (AP2/ERF) family play important roles in plant growth, development, and responses to biotic and abiotic stresses, we performed a functional characterization of *MtERF1* gene. Real-time PCR analysis revealed that *MtERF1* is mainly expressed in roots and is inducible by NaCl and low temperature. Additionally, under salt stress, a greater increase in the expression of *MtERF1* was found in TN1.11 plants than that in TN6.18. Therefore, the *MtERF1* pattern of expression may provide a useful marker for discriminating among lines of *M. truncatula* and can be used as a tool in breeding programs aiming at obtaining *Medicago* lines with improved salt tolerance.

## 1. Introduction

Due to the unpredictability of environmental conditions and the inability of plants to move in order to avoid unfavorable conditions, a number of abiotic stress factors threaten plant productivity and sustainability [1,2] Salinity is one of the main environmental stressors limiting crop production globally [3], it causes oxidative damages, ion toxicity, and nutrition imbalance [2,4,5,6]. According to the United Nations (UN) Environment Program (UNEP), worldwide, approximately 50% of agricultural lands are now characterized as saline soils [6]. Additionally, this area increases every day due to inadequate irrigation practices and it aggravates the salinity problem [7].

Survival under this stress requires the integration of adaptive metabolic, physiological, and molecular responses. In response to salinity, plants employ different strategies and mechanisms to accumulate organic solutes in the tissue, and provide tissue tolerance to high salt concentrations [7,8,9,10] These organic solutes can protect plants against short term and high intensity salt stress [11]. Moreover, it is known that soluble protein content is an important indicator of physiological status of plants [12]. Thus, understanding the different adaption mechanisms to environmental stresses may lead to novel strategies for plant improvement.

According to the ability to grow on high salt medium, plants have been classified as glycophytes or halophytes, where glycophytes cannot grow well under salt stress condition whereas halophytes grow well under high salinity. Most crop species are glycophytes and cannot tolerate salt stress [13,14], so to cope with this stress, plants have evolved complex mechanisms and elaborated signaling network that perceives signals from their surroundings and appropriately responds to environmental changes by modulating the expression of responsive genes [15]. These genes encode two major groups of proteins: functional and regulatory proteins. The main regulators of abiotic stress mediated gene expression are transcription factors (TFs), which have the capacity to recognize and bind specifically to cis-elements in the promoters of stress-responsive genes, and regulate their transcription [15,16,17], TFs function as terminal transducers and directly modulate gene expression of an array of downstream genes [18,19,20,21] Due to this property, the manipulation of TFs is a very useful strategy for imparting multiple stress tolerance in plants [22,23,24]. The APETALA2/ethylene responsive factor (AP2/ERF) superfamily is one of the largest groups of transcription factors in plants [25]. This family is characterized by the presence of a common domain of about 60–70 amino acids residues known as the AP2 domain. A simple classification based on the copy number of AP2 domains yielded four families: AP2, ERF, RAV and soloist [26,27]. The AP2 family owns duplicated AP2/ERF domains, whereas the ERF family exhibits a single AP2/ERF domain, the RAV family has one B3 domain and one AP2/ERF domain and the Soloist family contains a small group of TFs with a highly divergent single AP2 domain (AP2-like domain) and gene structure.

The ERF family is further subdivided into the ERF and dehydration responsive element binding proteins (DREB) subfamilies on the basis of the similarities in amino acid residues of the AP2 domain [28]. The TFs from the ERF and DREB subfamilies, are closely associated with responses to environmental stress, such as pathogen and disease stimuli [28,29], salinity [30,31], drought [31], and freezing [32,33]. With more extensive plant genome sequences, AP2/ERF gene families have been identified in various plants, such as Arabidopsis [34], soybean [35], rice [36], potato [25], *Medicago truncatula* [37], barley [38], and *Ammopiptanthus nanus* [39], among others.

Moreover, legumes are the plants in family Fabaceae or Leguminosae, which are playing a crucial role in crop rotation due to their symbiotic nitrogen-fixing bacteria in structures [40,41]. Fabaceae are primarily grown for human consumption, for livestock forage and silage, and as soil-enhancing green manure [42,43]. Forage crops are the backbone of sustainable agriculture; they are often grown in less favorable areas and thus require sophisticated protective mechanisms to withstand severe environmental conditions [43]. The transcription factors play a crucial role in enhancing some legume species to adapt during abiotic stresses. Thus, we have been interested in characterizing certain transcription factor genes from *M. truncatula*, which is an omni-Mediterranean forage legume species and a model plant for legume biological studies in view of its small diploid genome, self-fertile nature, relatively short life cycle and high genetic transformation efficiency [42,44]. Due to these characteristics and to the fact that it is a close relative of alfalfa and clovers, *M. truncatula* has become the focus of intensive research around the globe, aimed at identifying and characterizing major stress responsive genes using modern tools, such as genomics as well as genetic transformation [44,45,46].

This study aims to (i) analyze the morpho-physiological and biochemical responses of four *M. truncatula* lines under salt stress, and (ii) to explore the expression of an ERF gene in *M. truncatula* under salt stress.

## 2. Results

### 2.1. Morpho-Physiological Responses

Results from ANOVA showed that the variation in measured traits were explained by the effects of line, treatment and the interaction of line × treatment (Table 1). The maximum effect was observed for the line factor. There were clear treatment and line effects on most of the traits. However, the variation of only six traits was explained by the interaction line × treatment. The traits include the length of stems, number of leaves, aerial fresh weight, aerial dry weight, root fresh weight, and root dry weight.

Salt stress significantly decreased the fresh and dry biomass of shoots and root organs of *M. truncatula*. Salinity stress was associated with 49%, 82%, 18%, and 86% decrease in shoot fresh weights while the shoot dry weight was reduced by 40%, 76%, 31%, and 68% in A10, JA17, TN1.11, and TN6.18 under salt stress, respectively. Similarly, compared to the control plants, the root fresh weight was reduced under salt stress by 61%, 66%, 29%, and 29% while the reductions in root dry weight were 93%, 76%, 30%, and 33% in A10, JA17, TN1.11, and TN6.18, respectively, in salinity treated plants (Table 2). In comparison to controls, the effect of salt stress on plant biomass was much more noticeable for TN6.18; which it had the least effect on root fresh weight and the biggest effect on aerial fresh weight.

Under salt stress, the length of stems was reduced by 36%, 46%, 14%, and 56% in the A10, JA17, TN1.11, and TN6.18 lines, respectively.

**Figure 1:**
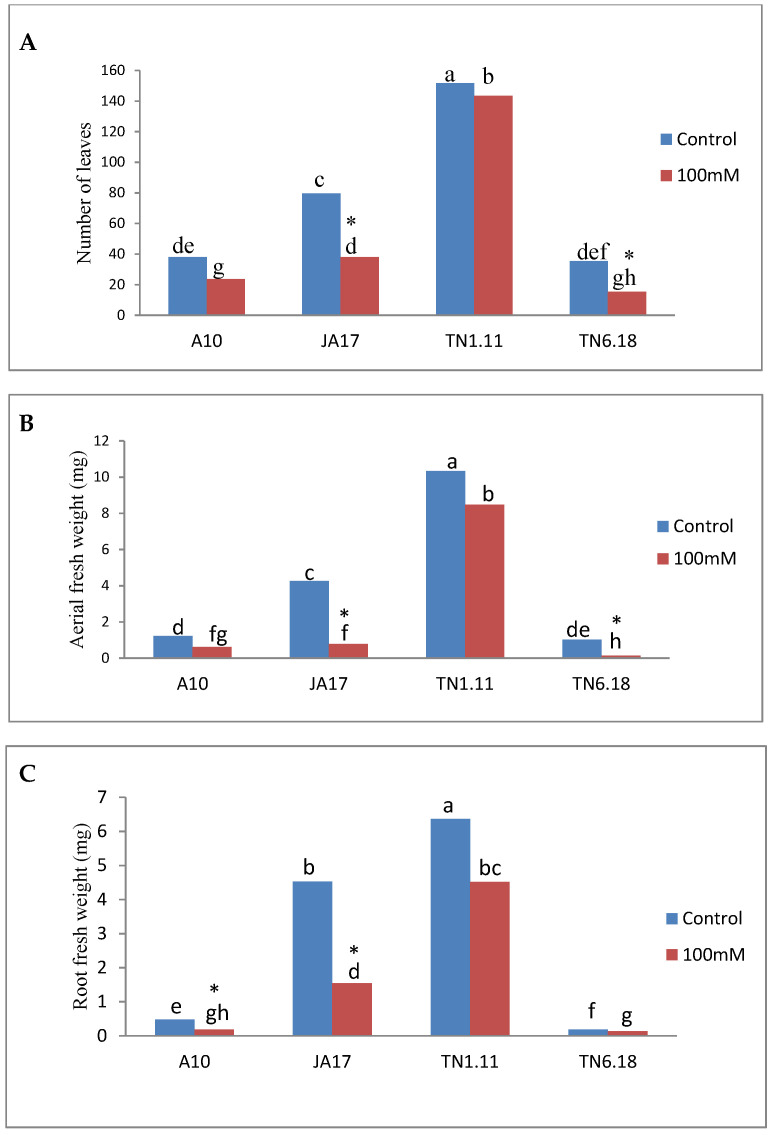
Means of (**A**) leaves number, (**B**) aerial fresh weight, and (**C**) root fresh weight for the four lines of *M. truncatula* under control treatment and 100 mM NaCl. Different letters set in bold represent significant differences among treatments as tested using a Duncan’s test. Asterisks indicate tests are taken as significant at *P* ≤ 0.05.

Salinity stress significantly (*P* ≤ 0.05) reduced the number of leaves in JA17 and TN6.18 lines (Figure 1).

All the measured traits for the four lines showed high heritability values (*H*^2^ > 0.4). The broad-sense heritability (*H*^2^) values of the traits ranged from 0.90 to 0.97 and from 0.91 to 0.99 under the control treatment and salt stress, respectively (Table 2).

Among the 30 possible correlations, 25 were significant and were positive (Table 3). Only the length of stems was not significantly correlated with any of the traits in the control treatment.

#### Principal Component Analysis and Clustering Analysis

The first three principal components with eigenvalues > 1 explained 100% of the total variation among the studied genotypes grown under salt stress. The first two axes explained 87% of the total phenotypic variation. The first axis was mainly correlated to the number of leaves, the aerial fresh weight and the aerial dry weight. The second axis was explained by the root dry weight. The distribution of the studied genotypes on the first two axes of principal component analysis (PCA) showed an important genetic variation.

**Figure 2:**
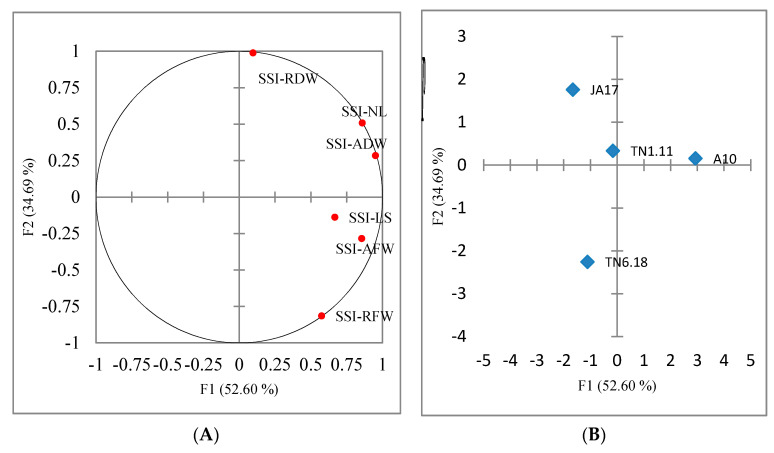
Principal components analysis with traits recorded under 100 mM NaCl condition for 4 lines of *M. truncatula*. (**A**) Contribution of traits to the first two principal component analysis (PCA) axes. Length of stems (SSI-LS), number of leaves (SSI-NL), aerial fresh weight (SSI-AFW), aerial dry weight (SSI-ADW), root fresh weight (SSI-RFW), root dry weight (SSI-RDW). (**B**) Distribution of the four lines on the first two PCA axes.

The positive side of the PCA gathered the tolerant and the moderately tolerant lines (A10, TN1.11, and JA17), which were marked by high values of salt sensitivity index SSI (Figure 2B). However, the negative side of the PCA was associated with the sensitive line TN6.18.

**Figure 3:**
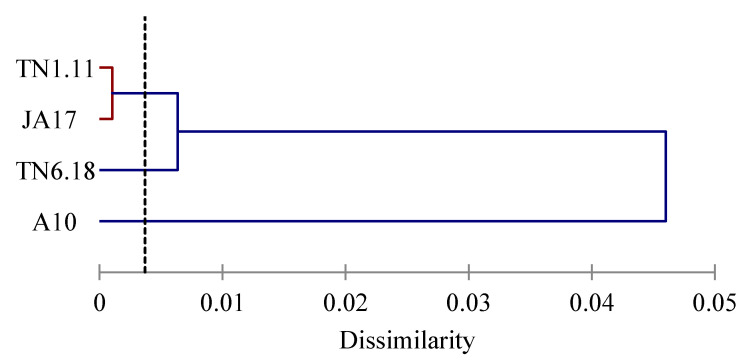
Dendrogram of the lines of *M. truncatula* based on Euclidean distances of dissimilarity matrix using the Ward’s method.

The lines were clustered into three groups (Figure 3). The first group was formed by the tolerant line A10, the second group was constituted by the two moderately tolerant lines TN1.11 and JA17, and the third group was composed by the sensitive line TN6.18.

### 2.2. Biochemical Responses

Salt stress influenced soluble sugars, proteins and malondialdehyde (MDA) content (Table 4). There were clear treatment and line effects on the soluble sugar. However, the effect of treatment was not significant for proteins and MDA content.

**Figure 4:**
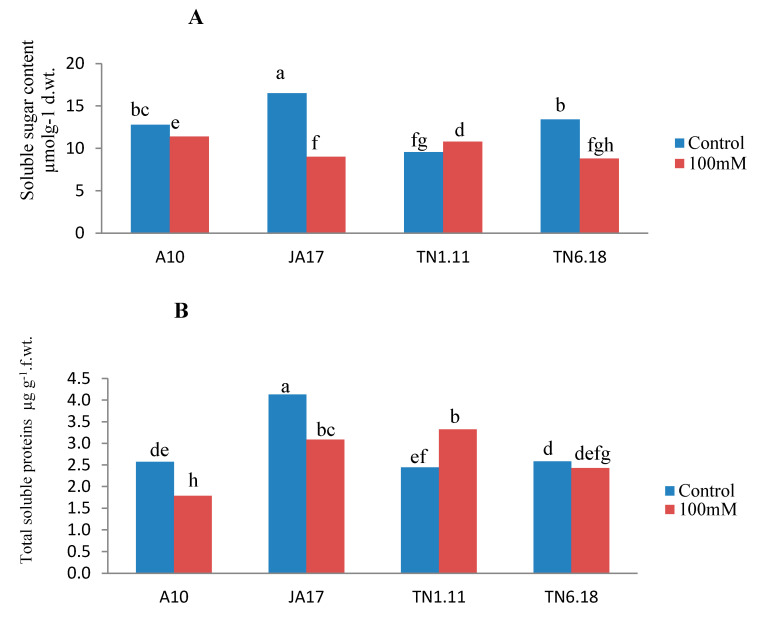

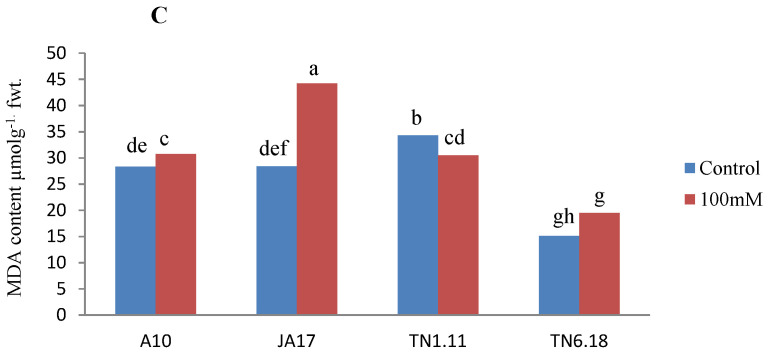
Variability of soluble sugar (**A**), total proteins content (**B**), MDA content (**C**) in A10, JA17, TN1.11, and TN6.18 lines of *M. truncatula* under control treatment and 100 mM NaCl. Means with the same or common letters are not significantly different among studied lines as tested using a Duncan’s test.

Under salt stress, there was a decrease of sugars and protein content in all the studied lines except for TN1.11 (Figure 4). This decrease was more pronounced for the soluble sugars (45%) and soluble proteins (30%) for the lines JA17 and A10, respectively. Compared with the control treatment, MDA content increased as a result of salt stress for all lines except TN1.11, indicating enhanced lipid peroxidation. However, the highest increase was observed in JA17 (54%) (Figure 4). Protein and soluble sugar content accumulate in TN1.11 subjected to salinity stress conditions to confer stress tolerance to this line.

### 2.3. Molecular Responses

#### 2.3.1. Gene Expression Analysis

To study the role of *MtERF1* in salt stress response, we examined the expression of *MTERF1* in 21-day-old lines grown on a mixture of sand and compost (3:1. *v*/*v*) treated with 100 mM NaCl for 30 days. ANOVA showed that the variation in salt stress response was explained by the effects of line, tissue treatment, the interactions line × tissue, line × treatment, tissue × treatment, line × tissue × treatment. The maximum effect was observed for the treatment factor (Table 5).

**Figure 5:**
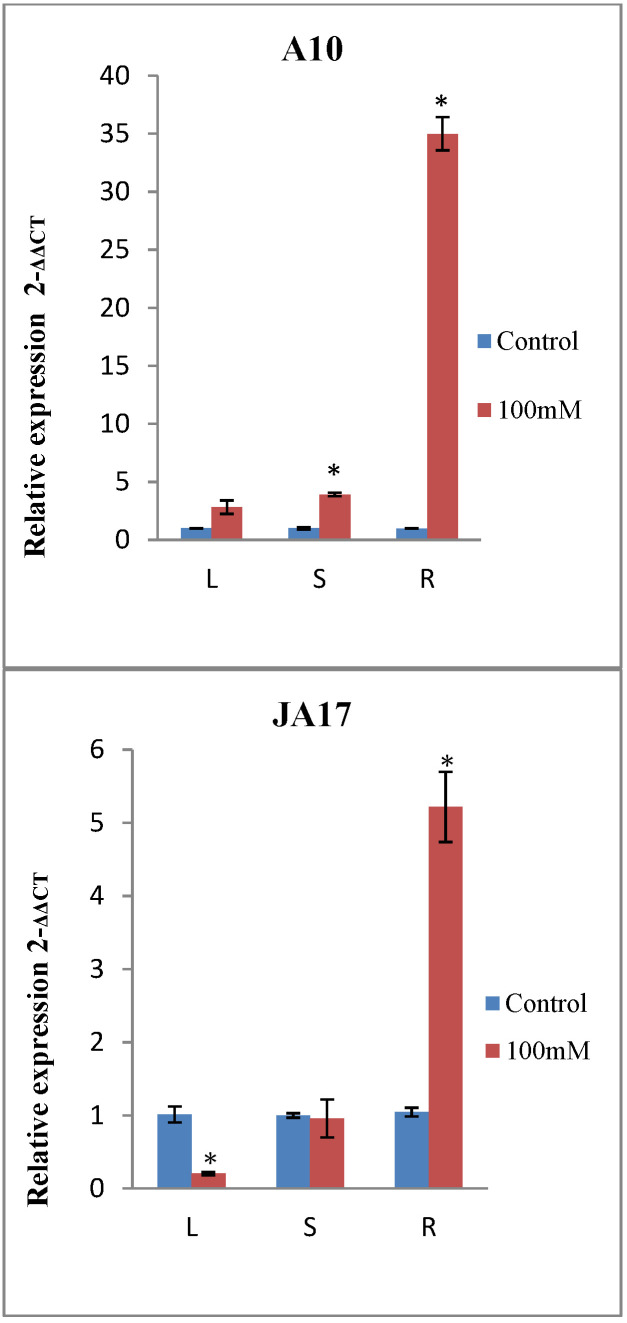

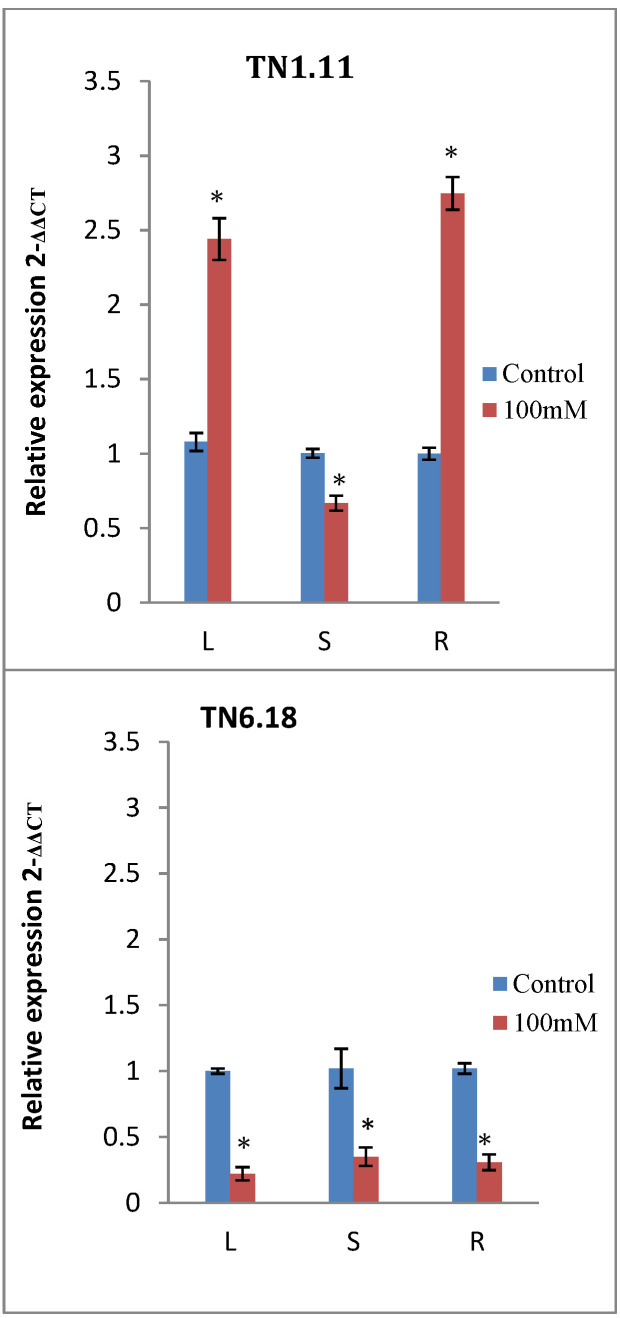
Expression analysis of *MtERF1* in different tissues of the four lines of *M. truncatula* under control treatment and 100 mM NaCl. Leaves (L), stems (S) and roots (R). Asterisks indicate significant differences between treatments as estimated by ANOVA (* *P* ≤ 0.05).

The spatial expression pattern of *MtERF1*, was determined by analyzing the expression profiles of *MtERF1* in three different organs: roots, stems, and leaves by qRT-PCR. Results showed that *MtERF1* can be detected in all tissues of *M. truncatula,* but with different expression levels (Table 6). *MtERF1* was mainly expressed in roots and the leaves. The highest induction was observed in roots under salt stress in all lines except in the sensitive one (TN.6.18) (Figure 5). In stems, only A10 showed a significant expression profile of *MtERF1,* and this may explain why only the length of stems was not correlated with any morphological traits.

**Figure 6:**
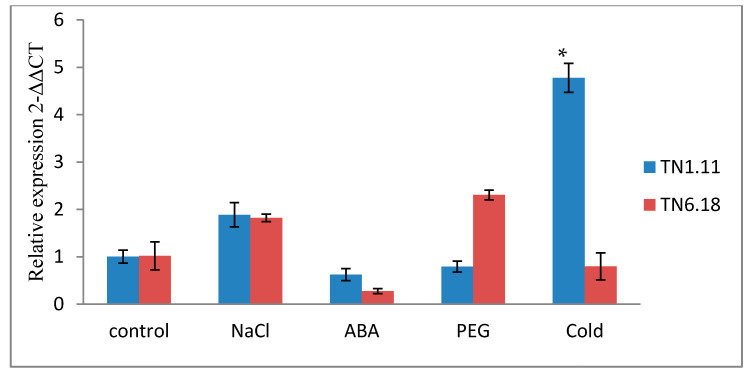
Expression analysis of *MtERF1* of two lines of *M. truncatula* TN1.11 and TN6.18 under different treatments: control, 200 mM NaCl, 10 µM ABA, 20% PEG, and cold 4 °C after 24 h. A representative example out of three biological replicates is shown, the error bars signify standard error and the asterisks as estimated by ANOVA (* *P* ≤ 0.05).

**Figure 7:**
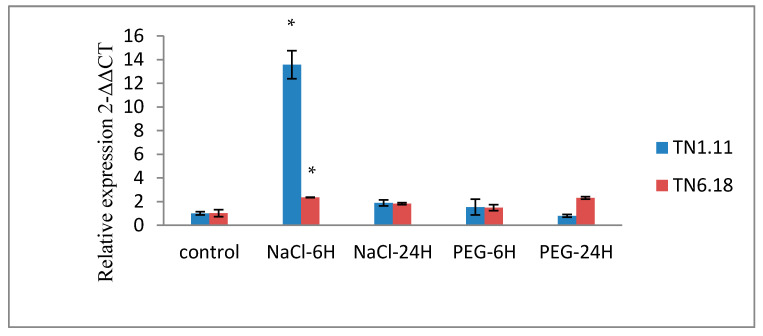
Expression analysis of *MtERF1* of two lines of *M. truncatula* TN1.11 and TN6.18 under different treatment: control, 200 mM NaCl and 20% PEG after 6 and 24 h. The error bars signify standard error in data from three independent experiments and the asterisks as estimated by ANOVA (* *P* ≤ 0.05).

To determine the short-term response of *MtERF1* to salt stress, we analyze the relative transcript levels after 6 and 24 h (Figure 6 and Figure 7). The abundance of *MtERF1* increased about 14 times by salt treatment compared with the control after 6 h (Figure 7).

For the line A10, *MtERF1* gene expression showed a significant increase in stems and roots under NaCl treatment, but the highest level of expression was detected in roots (Figure 5).

As shown in Figure 5, salt treatment upregulated the expression of *MtERF1* in leaves and roots for the line TN1.11, while a downregulation was observed in the stems.

In the case of JA17, *MtERF1* gene expression decreased by the NaCl treatment in leaves, whereas a sharp increase was observed in roots.

For TN6.18, a strong reduction in the expression of *MtERF1* in all examined tissues was observed by the application of salt treatment suggesting that TN6.18 was more sensitive to salt stress than the others lines (Figure 5).

#### 2.3.2. Expression Analysis of MtERF1 under Abiotic Stresses and ABA Treatment

The expression profiles of *MtERF1* by qRT-PCR showed a significant induction by cold and salt treatment (200 mM) for the tolerant line TN1.11 (Table 7) after 24 h for cold treatment (Figure 6) and after 6 h for salt treatment (Figure 7), which mean the implication of *MtERF1* in early salt stress responses by providing initial protection and amplification of signals. However, under the 20% PEG treatment, the upregulation of *MtERF1* was only detected for the line TN6.18, but it was not significant. Moreover, for the 10 µM ABA treatment, the expression levels were downregulated for both lines after 24 h (Figure 6).

As shown in Figure 7, the amounts of the *MtERF1* transcripts significantly increased after 6 h of 200 mM NaCl treatment in both lines. However, they increased to 14-fold higher than the control plants grown under normal conditions for the tolerant line TN1.11. Hence, the result indicates a rapid response of *MtERF1* to salt stress because the accumulation decreases after 24 h of salt treatment.

Taken together, these results suggest that TN1.11 is a salt-tolerant line of *Medicago truncatula* and *MtERF1* may play a role in regulating root growth and could be a marker gene for salt tolerance.

## 3. Discussion

### 3.1. Morphological and Photosynthetic Characteristics Variation

Reductions in the biomass under salt stress were indicative of severe growth limitations. Salinity had many effects not only on the biomass, but also on other morphological parameters, such as plant height, number of leaves, and root length salinity was reported to reduce shoot and root weights [47,48,49,50,51,52,53]. Similarly, in the present study, we recorded reduced growth during stress conditions. Our results revealed that the 100 mM NaCl stress treatment has caused reduction of the biomass in all lines, but more pronounced reduction was found for the sensitive line TN6.18.

The broad-sense heritability (*H*^2^) for the traits measured showed high values. This high heritability found for most analyzed traits may be explained by a large genetic variance rather than by a smaller environmental variance, as already was suggested by Barton and Turelli (1989) [54] and by Badri et al. (2016) [55]. Fitness components are generally less heritable than morphological or physiological characters [56].

### 3.2. Biochemical Characterization

At the physiological level, accumulation of osmolytes acting as osmoprotectants, such as proline, glycine betaine, soluble proteins, and soluble sugars, is a strategy to overcome osmotic stress provoked by salinity [57,58]. These osmoprotectants are essential to maintain cellular osmotic balance, detoxification of reactive oxygen species, maintenance of membrane integrity and stabilization of proteins [59].

In our study, two osmoprotectants (soluble proteins and soluble sugars) were measured in leaves. The line TN1.11 was found to be more salt tolerant than other lines. This was reflected by the increase in soluble proteins and soluble sugar. The increase in soluble protein content under stress may be the result of enhanced synthesis of specific stress-related proteins [59]. Furthermore, soluble sugars act as important osmolytes to maintain the cell homeostasis [60].

MDA is the principal product of polyunsaturated fatty acid peroxidation, which acts as toxic molecule and biological marker of oxidative stress [61]. The amount of MDA represents the degree of cell membrane damage under salt stress and is a common physiological indicator in evaluation of salt tolerance [61,62,63].

Previous studies have shown that the capacity to prevent membrane damage is correlated with the stress tolerance of plants [11,64]. The accumulation of MDA in salt treated *M. truncatula* implied clearly that the plants were suffering from stress. This high accumulation of MDA in *M. truncatula* made these plants more susceptible to oxidative damage under the conditions of abiotic stresses [65]. The low concentration of MDA and the stability of membrane integrity in TN1.11 suggest that this line was more protected against the oxidative stress than the other lines.

### 3.3. Expression Analysis of MtERF1 under Abiotic Stresses ABA Treatment

The AP2/ERF superfamily is one of the largest groups of the transcription factor family in plants which plays an important role in the regulation of plant development and tolerance to biotic and abiotic stresses [38]. It is considered as one of the most important families of gene regulators in plants [66].

ERFs, which contain an AP2 DNA-binding domain, form a plants specific superfamily of 123 transcriptional factors in *M. truncatula* [37], and play an important role in the transcriptional regulation of a variety of abiotic and biotic stress responses.

In previous studies, ERF subfamily gene expression levels were found to be higher than those of AP2 subfamily members, which may be related to the number of introns [67]. The gene expression rate is accelerated, leading to higher expression levels. when the number of introns is small [68]. Genes in the ERF subfamily are either devoid of introns or had only one to two introns [67]. In this study, a member of the AP2/ERF transcription factors, *MtERF1,* which is constituted of one exon, was studied under salt stress.

The *MtERF1* gene was inducible by salt treatment and cold, indicating the involvement of this transcription factors in abiotic stress responses, demonstrated previously in several studies [68,69,70,71,72].

Moreover, ERF1 was rapidly induced by salt stress within few hours (6 h) in the model legume *M. truncatula,* which is in agreement with previous studies realized in several plant species, such as *A. thaliana* [73,74,75,76], tomato [77], *L. japonicus* [78], and moss [79], which support the strong implication of TFs in early salt stress responses. It has been speculated that the early responsive genes may provide initial protection and amplification of signals [80].

Accumulating evidence indicates that AP2/ERF genes have different expression patterns in different organs, and play roles in regulating plant growth and organs development. In the current study, *MtERF1* expression was found to be most abundant in roots compared to leaves and stems, similarly to previous studies [36,79,81]. This organ/tissue-differentiated expression may be a consequence of differences in the existence/abundance of regulatory proteins that interact with cis-acting elements or other transcriptional/post-transcriptional regulators in the various organs/tissues.

### 3.4. MtERF1 Promoter Analysis of Cis-Acting Elements

**Figure 8:**
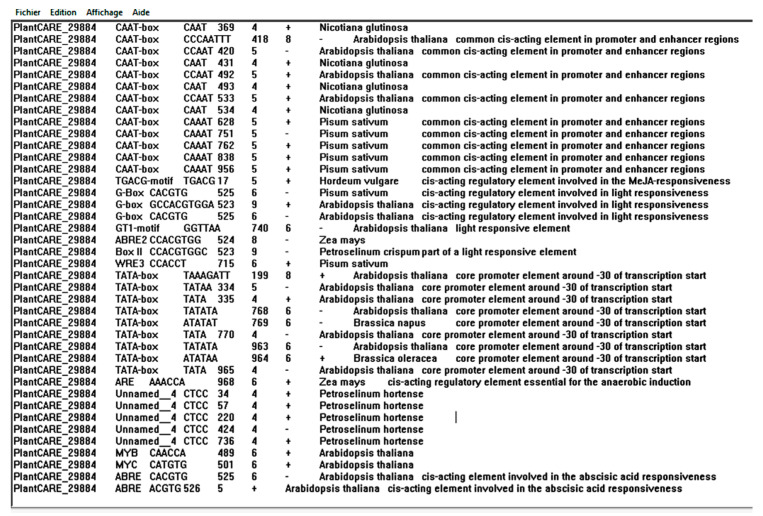
Some cis-acting elements identified in the promotor using the PlantCARE search tool.

Analysis of promoters provides useful information to understand the upstream transcription factors that govern the tightly controlled regulation of ERF genes [82]. In the past two decades, a large number of research studies on the regulation of ERFs have revealed a complex network of different transcription factors involved in their regulation, and the ERF promoters have been proposed to be the central hubs that integrate multiple environmental and internal developmental signals [83]. Our results showed that promoter regions of the *MtERF1* contained a certain number of TATA motif, CAAT motif, MYB, and MYC elements. We did not find obvious differences after ABA treatment although the promoter contained multiple ABREs and a large number of MYB/MYC binding sites, which can be explained by the fact that MYB and MYC factors act in an ABA-dependent manner at a later stage of stress responses to high-salt and water stresses and we had only analyzed the expression at 24 h (Figure 8) [83,84,85,86].

In brief, the large amount of stress-related cis-acting elements found in promoters of ERF subfamily genes supported their potential biological functions in regulating low-temperature stress and salt stress response in *M. truncatula*. In this study, qRT-PCR analysis of gene expression in response to ABA, PEG, cold and salt stresses showed that this gene did not respond significantly to ABA treatment, while it was induced by cold and salt stress and repressed by osmotic stress. These findings indicated that *MtERF1* plays an active role in responses to cold and salt stress. Furthermore, Gruber et al. (2009) [87] reported that several TF are induced at much higher levels by salt stress than by other treatments, suggesting certain specificity of the salt response, which is similar to findings reported in this study [88].

## 4. Material and Methods

### 4.1. Plant Material and Culture Conditions

Four lines of *M. truncatula* were used. They include the two Tunisian lines TN1.11 and TN6.18, the Moroccan line A10, lines belonging to a collection of genotypes used in the *M. truncatula* HapMap project, and Jemalong A17 (JA17) from the Australian collection, which was used to obtain the reference genome for *M. truncatula*. Three biological replicates were used in all analysis.

Seeds were scarified using sandpaper q60, then were kept for 72 h in the dark at 4 °C and thereafter transferred at 21 °C during 24 h for germination. The germinated seeds were sown into black pots with a two liters capacity filled with a mixture of sand and compost (3:1. *v*/*v*). Plants were grown in a growth chamber under controlled conditions at a temperature of 24 °C/18 °C (day/night), a relative humidity of 60–80%, and a photoperiod of 16/8 h. Plants were irrigated with Fahräeus nutritive solution [89] once every 2 days, and after 21 days they were randomly divided into two groups one for the control treatment and the other for salt stress (100 mM NaCl).

### 4.2. Morphological and Photosynthetic Parameters

Fourteen parameters were measured for the studied lines including the length of stems (LS, cm), length of roots (LR, cm), number of leaves (NL), number of axes (NA), aerial fresh weight (AFW, g), aerial dry weight (ADW, g), root fresh weight (RFW, g), root dry weight (RDW, g), root dry weight and aerial dry weight ratio (RDW/ADW), relative water content (RWC, %), water content (WC), and the relative growth rate (RGR), chlorophyll a (Chla, mg.g^−1^ FW), and chlorophyll b (Chlb, mg.g^−1^ FW) content.

The relative water content (RWC) was calculated as follows: RWC (%) = 100 [(LFW−LDW)/(LTW−LDW)], where LFW is the leaf fresh weight, LDW is the leaf dry weight, and LTW is the leaf turgid weight. Briefly, the fresh weight of a young leaf is determined. The leaves were left floating on distilled water, in Petri dishes, for 24 h and the turgid weight is then recorded. After that, the leaf tissues were dried in an oven at 70 °C for 24 h and their dry weight was measured.

Relative growth rate (RGR) or the mean relative growth rate was determined as the rate of increase in total dry weight per unit of plant weight according to Hunt (1982) thus:

RGR = (In W2 − In W1/t2 − t1).

RGR in g.g^−1^ day^−1^,

where W is the total plant weight (g), t is the time (days), and the subscripts 1 and 2 are initial and final harvest of biomass yield.

### 4.3. MDA Determination

The extent of lipid peroxidation was estimated by determining the concentration of malondialdehyde [90]. Briefly, 150 mg of fresh leaves were homogenized in TCA (0.1%) and centrifuged (13,000× *g*, 20 min) at 4 °C. Thereafter, an aliquot of the supernatant was added to 0.5% (*w*/*v*) TBA in 20% (*w*/*v*) TCA and the mixture was incubated at 95 °C for 30 min. The reaction was stopped by transferring tubes to ice for 10 min followed by a centrifugation step at 10,000× *g* for 10 min. The absorbance of supernatant was measured at 532 nm and the value for non-specific absorption at 600 nm was subtracted. The concentration of MDA was determined from the extinction coefficient of 155 mmol.L^−1^cm^−1^. Three independent extractions were made for each sample.

### 4.4. Measurement of Soluble Sugars Content

Soluble sugar content was quantified the method described by Yemm and Willis (1954) [91]. Briefly, 25 mg of dry weight of leaves was homogenized with 5 mL of 80% ethanol for 30 min at 70 °C. The extract was separated by centrifugation at 6000× *g* for 15 min. A second extraction was carried out under the same conditions as above. Then, the soluble sugars content of the aqueous extract was determined using the sulfuric acid anthrone colorimetric method. The soluble sugar content was revealed through absorbance measurement at 640 nm. The standard curve was drawn using glucose (1 to 14 µg/mL).

### 4.5. Measurement of Soluble Proteins

Fresh leaf material (150 mg) was homogenized with 2 mL buffer (1 M Tris-HCl pH8, 0.5 M EDTA, 100 mM PMSF. The homogenate was then centrifuged for 30 min at 12,000× *g* at 4 °C. The protein concentration in the supernatant was measured by the protein assay using BSA as a standard [92].

### 4.6. Gene Expression Assay by Quantitative Real-Time (RT-qPCR)

The coding sequence of MtERF1 was identified from the National Center for Biotechnology Information (NCBI) website (http://www.ncbi.nlm.nih.gov/ (accessed on 15 August 2016)), its accession number is XM_003607935. Then this sequence was blasted against the *M. truncatula* genome with expected values ≤ (1 × 10^−5)^. Finally, these proteins were submitted to the NCBI batch web CD-search tool (http://www.ncbi. nlm.nih.gov/Structure/bwrpsb/bwrpsb.cgi, (accessed on 20 August 2016)). Pfam (http://pfam.sanger.ac.uk/ (accessed on 3 March 2021)), and SMART (http://smart.embl-hei delberg.de/ (accessed on 3 March 2021)) to confirm the presence and completeness of the AP2 domain and the primers were used to amplify the CDS regions of *MtERF1* and then it was sequenced to be sure if it is the right sequence.

To determine the expression pattern of *MtERF1* in *M. truncatula* we analyzed its expression in three different organs: roots, stem, and leaves by real-time quantitative PCR (qRT-PCR) using *MtERF1* specific primers. Total RNA was extracted from the different tissues of the four studied lines according to the procedures described by [93]. The concentration and purity of the total RNA were assessed with a NanoDrop-ND 1000 UV-Vis Spectrophotometer (NanoDrop Technologies, Auckland, New Zealand), using 1 μL of total RNA. RNA purity was estimated from the A260/A280 absorbance ratio. RNA samples were treated with recombinant RNase-free DNase I (Roche, Mannheim, Germany) for removing possible genomic DNA contamination. Then, 1 µg of each RNA extract was used to synthesize cDNA by using the iScript™. Reverse Transcription Supermix for RT-qPCR (Bio-Rad, Marnes-la-Coquette, France), according to the manufacturer’s protocol. Gene-specific primer pairs for RT-qPCR were designed with Primer 3 software (Whitehead Instutute for Biomedical Researech, Cambridge, MA, USA). Each gene was evaluated at least in two independent runs. Each sample was amplified in biological triplicate by quantitative qRT-PCR using a Roche 2.0 Real-Time PCR Detection System with SYBR Green (Roche, Mannheim, Germany). The cycle program used was as follows: Initial denaturation at 50 °C for 2 min. then 95 °C at 30 s. followed by 2 cycles for 1 min at 95 °C. then 10 min at 95 °C followed by 40 cycles at 95 °C (10 s) and 60 °C (30 s) followed by 65 °C for 15 s. The amplification of the Actin gene was used as the normalization control. The mRNA fold difference was relative to that of untreated samples used as calibrator. The relative quantification value for *MtERF1* was calculated by the 2−ΔΔCT method.

### 4.7. Promoter Prediction and Analysis for MtERF1

In order to gain insight into the transcriptional regulation of *MtERF1* gene, putative stress- or hormone-responsive cis-acting elements were identified in 1 kb lengths of *M. truncatula* DNA sequence located immediately 5′ to the gene coding sequence using the PlantCARE search tool (http://bioinformatics.psb.ugent.be/webtools/plantcare/html/ (accessed on 15 August 2019)).

### 4.8. Treatment with Various Abiotic Stress

We examined the expression profiles of *MTERF1* genes by qRT-PCR as described above under ABA and abiotic stress treatment, including cold, NaCl, and PEG only for the two local lines TN1.11 and TN6.18.

The three additional abiotic elicitors which were used are: osmotic stress (PEG 20%), cold (4 °C) and ABA (10 µM). For the treatment with salt, PEG, and ABA, 21-day-old seedlings of *M. truncatula* were transferred to Fahräeus nutritive solution containing 200 mM NaCl or 20% PEG and incubated for 6 h and 24 h. For ABA treatment, the solution containing 10 µM ABA and incubated for 24 h. For cold treatment, 21-day-old seedlings of *M. truncatula* grown in soil were placed in a low temperature chamber at 4 °C for 24 h.

### 4.9. Statistical Analyses

A three-way analysis of variance was used to test for line, salt treatment differences, and line × treatment interaction effects. Only characters that showed a significant line × treatment interaction were used for further analysis. Means were compared using Duncan’s multiple range test at 5%. To find out whether or not traits are correlated to each other, Pearson coefficients were calculated. The significance level of associations between morphological traits and photosynthetic parameters was set to 0.05. All analyses were performed using SPSS software (SPSS Inc. Released 2007 SPSS for Windows. Version 16.0. Chicago, IL, USA).

Broad-sense heritability (*H^2^*) for each trait was estimated as described by Badri et al. (2016) [94].

*H*^2^ = σ^2^ g/σ^2^ g+ σ^2^ e

where σ^2^ g is the genetic variance observed between the lines and σ^2^ e is the environmental variance corresponding to the residual error between the eight replicates of the same genotype (=line).

To investigate the stratification of the lines, salt sensitivity index (SSI) for measured traits in lines of *M. truncatula* grown under salt stress were subjected to principal component analysis (PCA) and hierarchical cluster analysis (HCA). This analysis was carried out using XLSTAT software (Version 2014.5.03. Addinsoft, Paris, France).

## 5. Conclusions

Associations were discovered between exhibited phenotypes, lipid peroxidation, soluble proteins, and sugar accumulation, and gene expression. Salt tolerance seems to be related to lower MDA accumulation and a lower biomass reduction. Besides, accumulation of soluble proteins and soluble sugars was linked to salt-tolerance, being much higher for the tolerant genotype.

The expression pattern of *MTERF1* supports the fact that this gene is involved in mechanisms associated with salt tolerance and could be considered as a marker to discriminate *Medicago* genotypes with differing tolerance to salinity. Our expression profiling analysis of the *MtERF1* in various tissues and under different abiotic stresses should facilitate the identification of appropriate candidate genes for further functional characterization. The information obtained in this study showed that the *MTERF1* gene is strongly induced by salt in root tissues. Thus. *MTERF1* could be helpful in selecting candidate genotypes to be used by crop growers in salty areas or as progenitors in breeding programs for salt tolerance.

## Figures and Tables

**Table 1:** Effects of line, treatment and the interaction of line × treatment on measured traits for studied lines of *M. truncatula* under control treatment and 100 mM NaCl.

	Line (L)		Treatment (Treat)		L × Treat	
	F	*P*	F	*P*	F	*P*
NA	9.71	0.00	0.00	1.00	0.00	1.00
LS	235.88	0.00	146.72	0.00	7.40	0.00
NL	613.35	0.00	85.35	0.00	10.13	0.00
AFW	417.76	0.00	71.73	0.00	10.41	0.00
ADW	151.60	0.00	24.50	0.00	6.00	0.01
LR	22.15	0.00	15.80	0.00	1.63	0.22
RFW	116.57	0.00	30.97	0.00	8.79	0.00
RDW	86.44	0.00	6.79	0.02	14.30	0.00
Ratio	11.46	0.00	1.11	0.31	9.90	0.00
RWC	1.67	0.21	5.61	0.031	0.59	0.63
Chla	0.28	0.84	0.27	0.61	0.78	0.52
Chlb	1.17	0.35	1.15	0.30	0.19	0.90
WC	2.14	0.13	0.21	0.65	1.39	0.28
RGW	4.45	0.019	16.75	0.00	1.25	0.33

F is the coefficient of Snedecor-Fisher with significance at *P* ≤ 0.05. Number of axes (NA), length of stems (LS, cm), number of leaves (NL), aerial fresh weight (AFW, g), aerial dry weight (ADW, g), length of roots (LR, cm), root fresh weight (RFW, g), root dry weight (RDW, g), root dry weight and aerial dry weight ratio (RDW/ADW), root water content (RWC), chlorophyll a (Chla), chlorophyll b (Chlb), water content (WC), relative growth rate (RGW, g).

**Table 2:** Minimum, maximum, means, and broad-sense heritability (*H*^2^) of measured characters for the four lines of *M. truncatula* under control treatment and 100 mM NaCl.

Trait		Min.	Max.	Ave	F-Value	*P*	*H* ^2^
LS	Control	14.00	56.00	39.08	321.68	0.00	0.99
	Salt	6.00	45.00	26.00	70.89	0.00	0.96
NL	Control	31.00	154.00	76.17	535.55	0.00	0.99
	Salt	6.00	145.00	55.08	231.46	0.00	0.99
AFW	Control	0.79	10.96	4.21	294.19	0.00	0.99
	Salt	0.08	9.32	2.50	161.83	0.00	0.98
ADW	Control	0.08	3.60	1.05	59.06	0.00	0.95
	Salt	0.02	2.19	0.60	223.79	0.00	0.99
RFW	Control	0.14	7.24	2.88	106.01	0.00	0.97
	Salt	0.10	5.68	1.60	32.67	0.00	0.91
RDW	Control	0.01	0.93	0.41	28.60	0.00	0.90
	Salt	0.01	1.16	0.30	106.70	0.00	0.97

Minimum (Min.), maximum (Max.), means (Ave), coefficient of Snedecor-Fisher with significance at *P* ≤ 0.05 (F-value), and broad-sense heritability (*H*^2^).

**Table 3:** Matrices of correlations between measured traits for the four lines of *M. truncatula* under control treatment (down diagonal) and 100 mM NaCl (upper diagonal).

	LS	NL	AFW	ADW	RFW	RDW
LS	1.00	0.44	0.45	0.44	0.38	0.38
NL	0.75 *	1.00	1.00 *	0.98 *	0.94 *	0.82 *
AFW	0.74 *	0.98 *	1.00	0.99 *	0.93 *	0.80 *
ADW	0.72 *	0.99 *	1.00 *	1.00	0.91 *	0.79 *
RFW	0.65 *	0.94 *	0.93 *	0.94 *	1.00	0.95 *
RDW	0.69 *	0.98 *	0.99 *	0.99 *	0.95 *	1.00

* Significant correlation at 0.05 (bilateral).

**Table 4:** Effects of line, treatment and the interaction line × treatment on biochemical parameters for studied lines of *M. truncatula* under control treatment and 100 mM NaCl.

	Soluble Sugar		Proteins		MDA	
	F	*P*	F	*P*	F	*P*
Line	7.13	0.00	11.59	0.00	5.02	0.01
Treatment	48.69	0.00	0.22	0.65	0.24	0.63
Line × Treat	20.28	0.00	3.23	0.05	2.95	0.06

F is the coefficient of Snedecor-Fisher with significance at *P* ≤ 0.05.

**Table 5:** Effects of line, tissue, treatment, the interaction line × tissue, the interaction line × treatment, the interaction tissue × treatment, and the interaction line × tissue × treatment on the expression of *MtERF1* gene for studied lines of *M. truncatula* under control treatment and 100 mM NaCl.

Source	F	*P*
Line	403.38	0.00
tissue	426.99	0.00
treatment	540.28	0.00
Line × tissue	246.07	0.00
Line × treatment	405.85	0.00
tissue × treatment	424.58	0.00
Line × tissue × treatment	246.89	0.00

F is the coefficient of Snedecor-Fisher with significance at *P* ≤ 0.05.

**Table 6:** Expression analysis of *MtERF1* for the four lines of *M. truncatula* under control treatment and 100 mM NaCl.

	JA17		A10		TN1.11		TN6.18	
	F	*P*	F	*P*	F	*P*	F	*P*
Leaves	53.42	0.00	5.80	0.09	76.24	0.00	202.47	0.00
Stems	0.024	0.88	232.85	0.00	24.66	0.00	15.44	0.02
Roots	71.77	0.00	337.16	0.00	185.63	0.00	72.2	0.00

F is the coefficient of Snedecor-Fisher with significance at *P* ≤ 0.05.

**Table 7:** ANOVA test for the analysis of *MtERF1* of two lines of *M. truncatula* TN1.11 and TN6.18 under different treatment: control, 200 mM NaCl and 20% PEG (after 6 and 24 h), 10 µM ABA and 4 °C (after 24 h).

Treatment	TN1.11		TN6.18	
	F	*P*	F	*P*
NaCl-6 h	223.37	0.00	40.81	0.02
PEG-6 h	1.25	0.38	2.77	0.24
NaCl-24 h	6.17	0.13	2.66	0.20
PEG-24 h	0.01	0.95	8.75	0.06
ABA-24 h	1.74	0.26	2.54	0.19
Cold-24 h	57.27	0.00	1.24	0.35

F is the coefficient of Snedecor-Fisher with significance at *P* ≤ 0.05.
